# Exploiting SNP Correlations within Random Forest for Genome-Wide Association Studies

**DOI:** 10.1371/journal.pone.0093379

**Published:** 2014-04-02

**Authors:** Vincent Botta, Gilles Louppe, Pierre Geurts, Louis Wehenkel

**Affiliations:** Department of EE and CS & GIGA-Research, University of Liège, Belgium; The University of Chicago, United States of America

## Abstract

The primary goal of genome-wide association studies (GWAS) is to discover variants that could lead, in isolation or in combination, to a particular trait or disease. Standard approaches to GWAS, however, are usually based on univariate hypothesis tests and therefore can account neither for correlations due to linkage disequilibrium nor for combinations of several markers. To discover and leverage such potential multivariate interactions, we propose in this work an extension of the Random Forest algorithm tailored for structured GWAS data. In terms of risk prediction, we show empirically on several GWAS datasets that the proposed *T-Trees* method significantly outperforms both the original Random Forest algorithm and standard linear models, thereby suggesting the actual existence of multivariate non-linear effects due to the combinations of several SNPs. We also demonstrate that variable importances as derived from our method can help identify relevant loci. Finally, we highlight the strong impact that quality control procedures may have, both in terms of predictive power and loci identification. Variable importance results and *T-Trees* source code are all available at www.montefiore.ulg.ac.be/~botta/ttrees/ and github.com/0asa/TTree-source respectively.

## Introduction

Advances in genetic marker technology now allow for the dense genotyping of hundreds of thousands of single nucleotide polymorphisms (SNPs). It is now possible to create, at a moderate cost, representative samples counting thousands of individuals each characterized by up to a million of genetic markers spanning their whole genome. From these data, genome-wide association studies (GWAS) aim to discover variants spread over the genome that could, in isolation or in combination, lead to a particular trait or an unfortunate phenotype such as a disease. The basic idea behind a GWAS is to statistically analyze the genetic differences between two populations: healthy vs. affected individuals [1].

The standard approach to GWAS is based on univariate hypothesis tests, where the potential association of each genetic marker is assessed in isolation of the others through the computation of p-values based on statistical assumptions about the data distribution [1]–[3]. While this standard approach has been at the basis of many novel loci unraveled in the last years for several complex diseases, it has several intrinsic limitations: (i) it does not directly account for correlations among the explanatory variables, while in the context of GWAS this correlation is often very strong, because of linkage disequilibrium (LD) or artifacts induced by the experiment design; (ii) it does not account for genetic interactions, i.e. causal effects that are only observed when specific combinations of mutations and/or non-mutations are jointly present; (iii) it does not directly provide predictive models for the genetic risk.

Some of these limitations are specifically addressed by advanced multivariate statistical and machine learning techniques. Bayesian linear regression methods and mixed-effect models that were originally proposed in the context of genomic selection have been adapted for GWAS [4]–[7]. The main strength of these methods is the possibility to take into account population structure, confounding effects, and linkage disequilibrium through the incorporation of appropriate priors. Generic machine learning (ML) techniques have also been exploited and adapted for GWAS. From the ML point of view, a GWAS is a supervised classification problem defined by thousands of individuals partitioned into two output classes, and described by several hundreds of thousands of discrete input variables corresponding to the SNPs (each variable having typically three possible values – 0, 1 or 2 – representing the number of mutant alleles present for the corresponding SNP). In the literature, several classification methods have been applied on GWAS data, such as Support Vector Machines, Logistic or Penalized Regression, Neural Networks or Random Forests (RF) [8]–[10]. In particular, RF-like methods are very attractive in this context, as they have several intrinsic features that fit very well with the requirements of GWAS as a supervised learning problem [11]–[15]. First, they allow to build a predictive model without making any assumption about the underlying relationship between genotype and phenotype. Second, several variable importance measures can be derived from these models. In the context of GWAS, they may help identify genomic regions containing causal mutations which, in isolation but also in combination, may be associated with the studied phenotype. Third, they are computationally efficient and almost free of hyper-parameters that need to be tuned, making them easily applicable to very high-dimensional GWAS datasets.

Several works have shown the good performance of RF methods either on simulated [11] or on real GWAS data [13], and in comparison with other methods, such as classical bayesian regression methods proposed in genomic selection [16] or other supervised learning methods [17]. The ability of RF to detect interacting SNPs has been analyzed for example in [12], [14]. These studies show that RF performs better than univariate tests in this goal, as expected, but that the probability to detect interacting SNPs drops rapidly when the total number of SNPs increases. RF importance scores were used by several authors as a pre-filtering tool before the application of statistical tests to find interacting SNPs [18], [19]. The effect of LD on variable importance measures derived from RF models has been investigated for example in [13], [20]–[22]. It mainly results in a reduction or dilution of the importance of SNPs that are in LD with many other SNPs, which may hinder even the discovery of strong effects shared by several SNPs. Several approaches have been proposed to address this problem. The simplest approach consists in pre-selecting only SNPs not in LD before building the forest [13]. Based on a similar idea, [23] proposed to prevent two SNPs in LD to appear in the same tree in a forest and adapted importance scores so that the importance of a SNP is only computed from the trees where this SNP appears. Another approach proposed in [23] is to use haplotypes instead of SNPs to build RF models. Despite good performances on simulated data, [23] nevertheless recommend to use the original RF methods together with their modified importance scores on real GWAS datasets. In our previous work [24], we proposed to treat haplotype blocks instead of single SNP inside decision tree test nodes using a maximum likelihood estimation of the conditional probability that the observed haplotype block is drawn from the population of cases (resp. controls). The results obtained on simulated data provided only marginally better results than a direct application of RF on SNPs.

Our main contribution in this paper is a novel tree-based ensemble method – called *Trees inside Trees* (*T-Trees*) – that takes into account the correlation structure among the genetic markers implied by linkage disequilibrium in GWAS data. In essence, we propose to replace the univariate split functions used in the nodes of a decision tree by non-linear multivariate split functions of contiguous SNPs, themselves modeled as decision trees.

We validate and compare our method with the original RF method on both synthetic datasets (results not reported here) and real life datasets coming from the Wellcome Trust Case Control Consortium [25]. We found that T-Trees systematically yield both improved predictive accuracy and better identification of causal loci. We also compare tree-based methods with standard linear models, showing the superiority of the former over the latter. Through a large-scale empirical investigation, a second contribution of our paper is hence to provide a better understanding of tree-based ensemble methods in real-life conditions while, for computational reasons, most previous empirical studies have focused on small simulated and/or strongly filtered data. As a third contribution, we also highlight in this study the very strong effect of quality control procedures on the classifiers induced by RF-like methods, both in terms of predictive power and loci identification.

## Materials and Methods

In this section, we first briefly describe the decision tree induction algorithm as well as standard tree-based ensemble methods. We then present and motivate the proposed *T-Trees* algorithm and describe how it relates with these former methods. We proceed with a review of the linear models later used for validation and then conclude with a description of the experimental protocol and quality controls used within this study. Pseudo-code and implementation details are provided in Supporting Information S1.

### Tree-based Ensemble Methods

A classification decision tree [26] is an input-output model represented by a tree structure. Any node in the tree represents a subset of the input space, with the root node being the whole input space itself. Internal nodes of the tree are labeled with a binary test (or split) dividing the subset they represent into two disjoint subsets corresponding to their left and right sub-trees. Binary tests are usually univariate linear split functions of the form 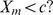, where 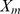 and 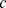 respectively denote the variable and the discretization threshold (or cut-point) used to partition the node into two subspaces. In the standard tree induction algorithm, combinations of 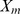 and 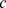 for all candidate variables and for all possible cut-points are typically investigated, and the one leading to the largest reduction of some impurity criterion is chosen to partition the node. Terminal nodes (or leaves) are labeled with a best guess value of the output variable, e.g. determined as the majority class in the subset represented by the leaf. The predicted output for a new instance is the label of the leaf reached by the instance when it is propagated through the tree by following the binary tests.

Single decision trees typically suffer from high variance, which makes them not very competitive in terms of accuracy. To circumvent this problem, ensembles of decision trees have been proposed and consist in growing a forest of 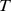 randomized trees whose predictions are aggregated (e.g., by majority voting) to form a final prediction. Representative algorithms of tree-based ensemble methods include Random Forests [27] or Extremely Randomized Trees ([28], Extra-Trees), and usually differ in the way they introduce randomization in the tree induction process. Random Forests (RF) exploit two sources of randomization: first, each tree in the ensemble is built on a bootstrap copy drawn with replacement from the original learning set; second, when splitting a node, instead of searching for the optimal binary test among all candidate variables 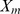, only a random subset of 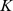 variables are investigated (while all possible cut-points for these variables remain considered). Using a random subset of variables typically leads to a better bias/variance trade-off and therefore to better performance (with respect to using all variables). By contrast, Extra-Trees (ET) do not use bootstrap copies to build each tree. As for RF though, it also only uses 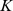 of the variables when splitting an internal node. However, instead of trying to the find the optimal cut-point, ET draws cut-points at random for each of the 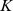 variables. From a statistical point of view, dropping bootstrap leads to an advantage in terms of bias, whereas the cut-point randomization has a variance reduction effect.

In this work, the hyper-parameters that are mainly considered are the number 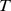 of trees and the number 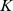 of variables investigated at each internal node. Because the higher 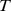, the better the performance, this parameter is usually set to the highest affordable value given the available computing resources. By default, 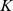 is often fixed to the square root of the total number of input variables. In some contexts (e.g., high output noise), it might also be advantageous to constrain the size of the trees within the ensemble, for example by setting a threshold 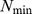 on the number of samples required to split a node or by globally limiting the number of test nodes within each tree. By default, however, trees are always fully grown.

As previously stated, tree-based ensemble methods can also be used to derive variable importance scores for each input variables. The two most used measures are the *Mean Decrease Impurity* (MDI) importance [27] and the *Mean Decrease Accuracy* (MDA) importance [27], [29]. The MDI importance of 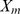 is computed as the weighted sum of the impurity decreases for all nodes where 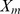 is used. It represents together with the importances of the other variables a decomposition of the information jointly provided by the inputs about the output [30]. The MDA importance of 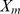 is the mean decrease in accuracy of the forest when the values of 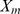 are randomly permuted in the out-of-bag samples. In this work, we choose the MDI importance for efficiency reasons. Indeed, unlike the MDA importance, it is embedded in the tree growing process and does not require any additional computation.

### Trees Inside Trees

In this section, we motivate and describe the proposed *T-Trees* algorithm as an extension of RF-like methods. We then proceed with a discussion of variable importance scores as they can be derived with our new method.

#### Motivation

Linkage disequilibrium in GWAS data reveals that tightly linked SNPs may sometimes be associated with each other. Mathematically, this suggests that input variables located in a same region may be structured in a non-random way. Despite evidence of this phenomenon, the structure of the feature space is almost never explicitly taken into account by standard data analysis tools. As such, we propose in this work a variant of tree-based ensemble methods that can exploit the local information carried by a region (or block) of genetic markers. The core principles of our method is to: (i) transform the original input space into groups of variables corresponding to contiguous and (potentially highly) correlated SNPs and (ii) replace the univariate linear split functions labeling the internal nodes of a decision tree by multivariate non-linear split functions of several SNPs located in a same block. In particular, we propose to model these complex binary tests as randomized decision trees built only from the SNPs of a block, hence making trees inside trees. From a machine learning point of view, our method relates to ensembles of oblique or functional trees [31], [32], which also replace the usual axis-aligned split functions by more complex alternatives. In the context of GWAS, potential benefits include:

- Capturing interactions between loci (i.e., blocks of SNPs). Since the size of the new feature space is inversely proportional to the size of the blocks, the greater is the chance of finding direct (resp. indirect) interactions between regions in consecutive nodes (resp. along a branch).
- Discovering SNP combinations (i.e., haplotypes or super-alleles) that are linked to the disease. Indeed, in standard tree-based methods, the chance of testing consecutively two SNPs from the same haplotype block is relatively small. With the proposed method, we force the exploration of such interactions when building the internal decision trees.
- Exploiting a group of surrogate variables. If two or more variables are in perfect LD, then they share the exact same information about the output variable and hence, due to the randomization in tree-based ensemble methods, each of these variables will be asymptotically equally selected in the ensemble, so that their respective importances will decrease as the number of surrogates increases [21], [23]. The ability to rank a block instead of a SNP will thus help identify a group of nearby highly correlated SNPs in association with the disease.

#### Algorithm

The first step of the algorithm is to transform the original input space, where each input variable corresponds to a SNP, to a new input space, where each input variable now encodes for a region of contiguous SNPs in the genome. As input, the method hence takes a *block map* defining how SNPs are grouped together. Such a map can be defined using available software for haplotype block identification [33] but then makes the proposed method strongly dependent on the output quality of such software. In this work, we consider instead to simply partition the set of SNPs into disjoint blocks of 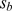 contiguous SNPs, where 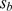 is an user-defined parameter controlling the size of a block.

In this new space, we then apply the standard RF algorithm but using the new group variables as input. In particular, this leads to two changes: (i) 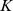 group variables are drawn at random when splitting an internal node (instead of drawing 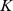 variables from the original space) and (ii) binary splits become multivariate non-linear functions of the SNPs in the corresponding block, and are themselves modeled as randomized decision trees. From these internal decision trees, numerical probability values of being a case can be derived for all samples in the node, and then finally used as a numerical attribute on which a cut-point can be fit (as in the standard RF algorithm) to partition the node.

In this work, internal trees are built as single Extra-Trees with internal parameter 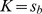 (found to be a good default value in [34]) and the following changes:

- The number of test nodes in an internal tree is limited by an *internal complexity* parameter denoted *IC*. The choice of this value depends on the nature of groups of variables. *IC* = 1 is an interesting choice for strongly correlated variables as they all carry the same information. Higher values are better suited when a combination of several variables is required to explain the outcome.
- Since the number of test nodes in internal trees is limited, expansion is done in random order to avoid tree degenerescence, as it would otherwise happen if nodes were expanded in depth-first order like in the standard algorithm.

**Figure 1:**
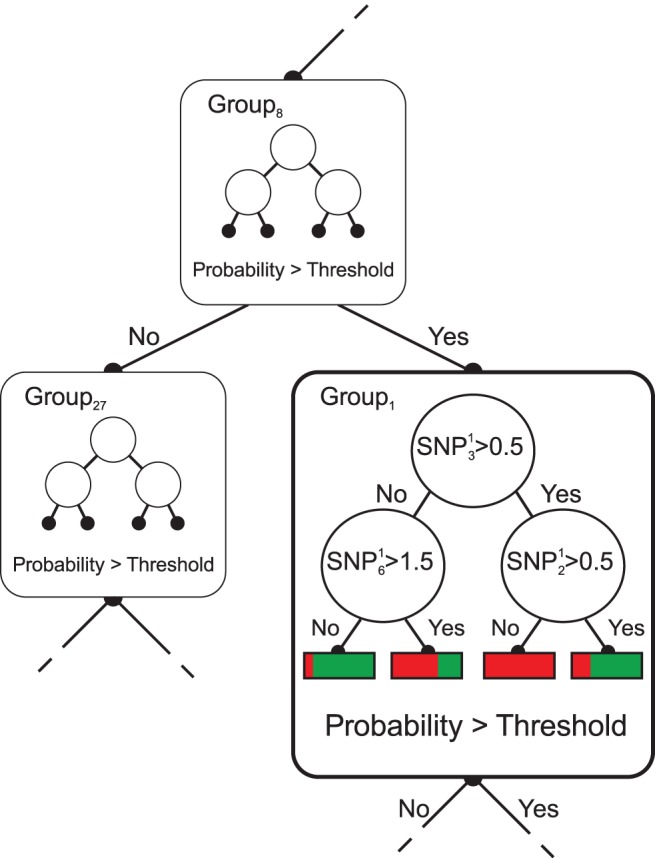
A closer look into a T-Tree test-node. The group 1 is tested. Out of this group, three SNPs are exploited by the weak learner. In red (resp. green), probability of being a case (resp. control) estimated by the weak-learner.

As an example, Figure 1 illustrates part of a single T-Tree. The large squared nodes are the *outer nodes*, while the small circled ones are the *inner nodes*, i.e. the nodes of the internal decision trees modeling the split functions of the outer nodes. The internal complexity 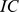 in this example has been set to 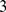. Hence, any internal tree counts at most 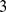 inner nodes. Inside the outer nodes, internal trees are expanded on groups of variables from the original input feature space. In particular, the internal tree in the highlighted outer node is expanded on group 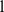, which means that its inner nodes are testing SNPs from this group only. For each object reaching the outer node, this small tree outputs a probability of being a disease case, and is used as a new numerical attribute on which a threshold is fit as in the standard RF method.

Finally, as in RF, an ensemble of 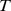 such T-Trees are grown on bootstrap copies of the learning set and their predictions are combined to form a final prediction.

#### Individual and group-wise importances

As for RF, T-Trees allow to derive variable importance scores. The nested structure of the model gives rise to several possible adaptations. In this work, we propose the following two MDI-like importance measures:

- *SNP importances*: For each individual variable (or SNP), its importance is computed as the weighted sum of the impurity decreases for all inner nodes where this variable is used (regardless of its originating group).
- *Block importances*: For each block of variables, its importance is computed as the weighted sum of the impurity decreases for all outer nodes where this group is used.

### Standard Linear Models

To be self comprehensive, we briefly describe in this section the standard linear methods with which we compare T-Trees. The first family of methods are log odds ratio approaches, as implemented in *PLINK v1.07* [4].

- *OR*: The log odds ratio approach assesses how the presence of a specific allele at a given locus increases or decreases the genetic risk, and additively combines these evidences into a global score. In particular, each individual is assigned with the average risk over non-missing SNP, defined as:

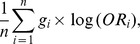
where 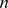 corresponds to the number of non-missing genotypes, 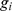 denotes the genotype (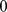, 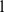 or 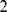) of 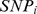 of the individual and 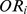 is the allelic odds ratio, that is the ratio between the proportion of cases having a specific allele and the proportion of controls having the same allele. In this variant of the method, odds ratios 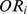 are directly derived from the dataset.

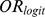
: Same as 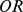, but 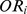 values are derived from a logistic regression model applied separately at each SNP 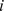 (and correspond to the 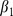 coefficient of the logistic regression model [35]).

The second family of methods are linear scoring functions (i.e., classifiers) of the form 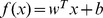, whose parameters 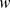 and 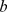 are obtained by minimizing.
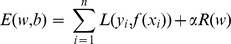
where 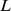 is a loss function that measures model fit and 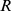 is a regularization term that penalizes complexity. All of them were evaluated using implementations from *Scikit-learn* [36].

- *SGD-L1*: Hinge loss function (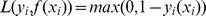) with 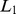 (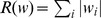) regularization, and optimized using a stochastic gradient procedure.
- *SGD-L2*: Same as SGD-L1, but using 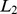 (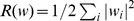) regularization.
- *Logit*: Logistic loss (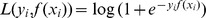) with 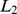 regularization.

Note that, as in tree-based methods, variable importance scores from linear models can also be derived, for example using the 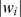 values.

### Datasets and Protocol

To validate our method, we performed experiments on the GWAS datasets made available by [25]. The WTCCC data collection contains 17000 genotypes, composed of 3000 shared controls and 14000 cases representing 7 common diseases of major public health concern: Crohn's disease (CD), bipolar disorder (BD), coronary artery disease (CAD), hypertension (HT), rheumatoid arthritis (RA), type 1 (T1D) and type 2 (T2D) diabetes. Individuals were genotyped with the Affymetrix GeneChip 500K Mapping Array Set and are described by about 500,000 SNPs (before the application of quality control filters).

In our experiments below, results on the Crohn's disease data are analyzed in more details. For this dataset, we investigate two different quality control (QC) filters that have been proposed in the literature. The first one corresponds to the original procedure described in [25], while the second one corresponds to the QC filter applied in ([37], Supporting Information S1). The second filter is quite stronger than the WTCCC filter. Datasets resulting from these filters are respectively denoted 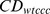 and 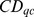. For the six other six datasets, we also consider the same filters but adapting the second one since it was not fully reproducible, as some filtering steps were tuned through undocumented visual inspections of various plots.

As evaluated methods do not deal with missing values as such, we chose to randomly fill the missing genotypes taking into account the genotypic distribution of the corresponding non missing values of the SNP over the corresponding joint cohort of cases and controls.

The predictive performance of all methods are assessed below by the area under the ROC curve metric (AUC), which is obtained on each dataset by 10-fold cross-validation (averaging the AUCs over the ten fold). All compared methods are evaluated on the exact same 10 folds to limit variability.

## Results

In this section, we present our results on 7 WTCCC datasets. We first compare T-Trees (TT) with RF in terms of predictive performance and then investigate in details the influence of the main parameters of our method (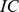 and 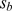) on the CD dataset. Next, we compare TT and RF with standard linear models. Finally, we evaluate and compare variable importance scores derived both from the TT and RF and discuss these results in the light of the loci confirmed in the literature.

### Predictive Power of TT vs RF

To make our comparison of RF and TT independent of the choice of their common parameters, we first carry out an exploration of the parameters 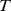, the number of trees in the ensemble, 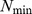, the minimal number of samples to split an (outer) node, and 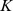, the number of SNPs or groups probed at each (outer) test node, both with RF and TT and on the 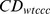 and 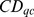 datasets. For TT, blocks of size 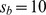 are used and the internal complexity is set to 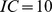. Table 1 reports the best performance of both methods, for various values of 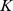, tuned parameter value for 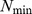 and 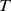 set to 1000. We observe that using T-Trees systematically and significantly improves the predictive accuracy, regardless of the dataset variant. Even with the lowest value of 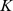, their AUCs are better than the best results of RF. Full results for various combinations of 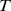, 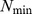 and 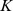 are presented in Figures S01–S04 in Supporting Information S1.

**Table 1:** Comparison between the two methods.

	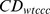		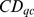	
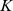	RF	TT	RF	TT
100	0.683	0.921	0.628	0.719
500	0.799	0.942	0.675	0.747
1000	0.845	**0.945**	0.684	**0.749**
2500	0.888	0.944	0.700	0.746
5000	0.909	0.938	0.698	0.740
10000	0.919	–	0.697	–
25000	0.907*	–	0.708*	–
50000	0.898*	–	0.698*	–

Predictive performance of RF and TT for different values of 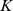, tuned value for 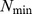 and 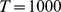. Best AUC values for each column are underlined; best AUC values for each dataset variant are shown in bold. For TT, 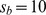 and 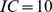. (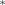 corresponds to RF with 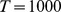 and 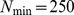); - TT was not applied for values of 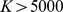; both for computational efficiency reasons.).

Trends observed in Table 1 are similar whatever the QC filter procedure but AUC scores are much lower with the stronger filter (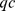) than with the other procedure (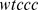). Notice that the optimal values of 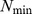 and 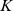 are also quite different for both methods: optimal results were observed with a slight pruning in the case of RF (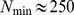) and a much stronger pruning in the case of TT (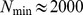); with RF, the optimal value of 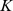 is around or above 10000, while the optimum is reached for 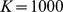 with TT. We also observe that TT is much less sensitive to the variation of 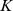 around its optimal value than RF is.

We now investigate the effect of block size 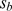 and internal complexity 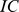. Table 2 summarizes results for different values of 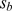 and 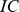 with 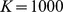 and 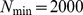 (which corresponds to the settings we identified as the optimal ones in the previous experiment). Results are consistent across the two datasets, as the optimal parameters for 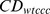 exactly coincides with the optimal parameters for 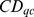. They also suggest that TT is quite robust against the block composition/choice. For both datasets, regardless of 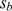 and the 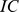, we indeed see that AUCs do not significantly fluctuate. The table even suggests that, no matter which block size is used, the only parameter that affects the predictive power is the internal complexity parameter (e.g., for 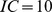, results for block sizes 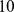 and 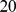 are almost identical). Nevertheless, we do observe a slight decrease in performance as 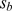 and 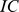 increase. We notice that larger block size and larger IC force T-Trees to explore a larger number of variables for each inner split, and we believe that by doing so they tend to overfit the training data.

**Table 2:** T-Trees: block map and internal complexity influence.

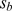	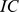	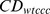	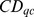
10	1	0.906	0.717
	5	**0.953**	**0.765**
	10	0.945	0.749
20	5	**0.955**	**0.755**
	10	0.945	0.740
	20	0.931	0.706
50	5	**0.938**	0.742
	10	0.937	**0.744**
	25	0.913	0.700

Effect of block size 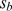 and internal complexity 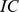, for 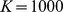, 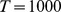, and 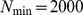. Maxima for each block size are highlighted in bold.

These strong and promising results suggest that using more than one SNP at each node improves tree-based classifiers. Table 3 however empirically ensures that our approach is effectively taking advantage of the structured nature of the variables (i.e., the LD pattern). When testing our approach with blocks of randomly positioned SNPs rather than contiguous SNPs (i.e., when breaking the surrounding LD structure), we indeed observe that performance is significantly worse, which suggests that the increased predictive power is not the sole consequence of the dimension reduction introduced by our methodology but is rather due to an effective use of an underlying structure. This result supports the effectiveness of our approach and confirms the initial intuition that led us to propose this method in the context of GWAS.

**Table 3:** T-Trees: contiguous versus randomized blocks.

	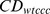		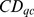	
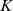	contig.	rand.	contig.	rand.
100	**0.903**	0.753	**0.690**	0.600
500	**0.936**	0.835	**0.728**	0.625
1000	**0.941**	0.853	**0.744**	0.627

Predictive performance of TT with 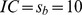, 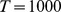 and 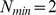, using **contig**uous blocks of 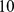 SNPs versus **rand**om blocks of 10 SNPs. Breaking the structure using randomized block maps drastically deteriorates the results.

Finally, Table 4 summarizes AUCs obtained on the six other disease related datasets, both with RF and TT and using the near optimal parameter settings identified on the CD dataset. As for the CD datasets, the TT systematically outperforms RF in terms of prediction accuracy (even when there is not much room left for improvement, i.e. when the AUC of RF is already very close to 1). No matter how the datasets were preprocessed, taking into account the structure of the descriptors allowed for a notable AUC increase, in all cases.

**Table 4:** Predictive power: auc comparisons on the six other WTCCC datasets.

	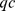		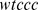	
	rf	tt	rf	tt
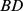	0.743	**0.813**	0.918	**0.959**
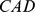	0.756	**0.814**	0.998	**0.999**
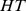	0.807	**0.866**	0.938	**0.969**
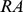	0.806	**0.830**	0.993	**0.996**
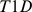	0.860	**0.870**	0.900	**0.940**
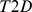	0.758	**0.834**	0.959	**0.979**

Predictive power of RF and TT on two variants of the 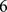 other wtccc datasets. The 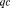 columns corresponds to the ''
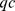
''-like filtered variant and the 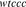 to the weakly filtered variant. (Parameters settings: RF: 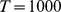, 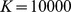, 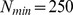; TT: 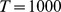, 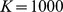, 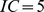, 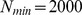).

The weaker QC filters of the WTCCC allowed both types of forests to reach unexpectedly high AUCs. In particular, for CAD and RA, RF and TT were able to almost perfectly predict individual disease statuses. On these two datasets, we notice that the removal of suspicious variables (i.e., showing a strong deviation from the Hardy-Weinberg equilibrium (HWE)) decreases the predictive power. In the most extreme case, with RF on CAD, AUC dropped from 0.998 to 0.756.

### Comparison with Linear Models

Next we compare tree-based algorithms with several standard linear models, as described in Materials and Methods. We focus here on predictive accuracy, and defer the comparison of SNPs rankings obtained with tree-based methods and linear models to the next Section.

Table 5 compares the predictive power of RF and TT with standard linear methods on the 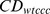 and 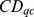 datasets. All linear methods were evaluated following the same 10-fold cross-validation scheme as for RF and TT and using the same folds. The regularization parameter 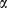 was chosen for each method so as to maximize the reported AUCs (Leading to 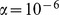, 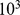, and 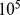 on 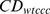 and 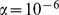, 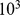, and 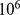 on 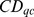, respectively for SGD-L1, SGD-L2, and Logit). Results for TT and RF correspond to the best results reported in Tables 1 and 2. Like for RF and TT, higher AUC values are reached on 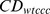 with the linear models. However, unlike for RF and TT, the difference between the two datasets is now very slight. For both datasets, the best results are achieved with T-Trees. While the difference is notable on 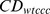, the gap between the best linear model and the best tree-based method is much smaller on 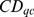. 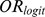 is the overall best linear model. Its good performance suggests that a notable part of the genetic risk can be explained as a linear combination of the individual SNPs odd ratios. The three regularized models are very close to each other, with a slight advantage for 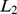 regularization (SGD-L2 and Logit) over 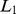 regularization (SGD-L1). These methods are however one step behind the log odds ratio methods, which might be explained by the very high dimensionality of the task and hence the less effective optimization procedure. Also, the fact that variables are considered independent of each other in the 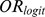 method makes it very robust against overfitting. Small noise effects due to a large number of irrelevant markers indeed tend to cancel each other on average.

**Table 5:** Comparison of the predictive power of tree-based methods and linear models.

	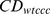	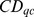
RF	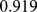	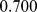
TT	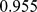	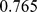
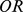	0.661	0.648
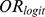	0.739	0.729
SGD-L1	0.623	0.613
SGD-L2	0.643	0.635
Logit	0.648	0.638
Poly [10]	0.716	–
LassoSVM [10]	0.762	–

As an additional comparison, we also report in the last two rows of Table 5 the best AUCs obtained by Abraham et al. in [10]. The first method, called Poly, is equivalent to the OR method with the difference that SNPs with high p-values (as computed on the training fold) were removed from the model. The second method, LassoSVM, trains a linear model by optimizing, with coordinate descent, square hinge loss with L1+L2 regularization. These AUCs were obtained by 10-fold cross-validation on a dataset that should be very close to our own 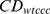 dataset. Although the QC filters might be slightly different from ours, we believe that these results can be compared with ours. We note from this comparison that better AUCs can be achieved with the OR method when filtering SNPs on the basis of their p-values. We applied the same filtering in combination with our OR method and indeed, AUC increased from 0.661 to 0.71 on 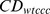. The AUC on 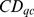 however only very slightly increased from 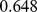 to 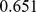 when filtering SNPs. The Lasso SVM method of [10] works much better than both the SGD-L1 and SGD-L2 methods, although its AUC is still inferior to that of the two tree-based ensemble methods, RF and TT.

### Variable Importance Analysis

We focus in this section on the analysis of variable importances on the Crohn's disease dataset, motivated by the availability of reported loci for this specific phenotype. In particular, we compare loci found by tree-based methods to loci reported in [25] and to the 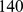 loci reported more recently in [37]. Similar results on the six other WTCCC datasets are provided online.

We computed SNP importances using both RF and T-Trees. Information related to the 200 first SNPs is summarized in Table 6 for the 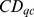 dataset. More detailed results are provided in Supporting Information S1 for 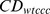 and 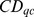 (see Tables S02 and S01 in Supporting Information S1). As several SNPs in these lists are close to each other, we grouped together contiguous SNPs that are separated by at most 20 SNPs in the dataset and only reported in Table 6 those SNP groups containing more than two SNPs, except when one such isolated SNP was highlighted in [25]. This procedure yields 16 regions and 4 isolated SNPs for RF and 27 regions and 4 isolated SNPs for T-Trees.

**Table 6:** Regions highlighted from the top 200 SNPs according to SNP importances with RF and T-Trees on *CD_qc_*.

Random Forests				
chr	size	rsid	trend p-value	importance
1	10	rs11209026^1,4,5^ (*IL23R*)	8.24 · 10^−18^	1.40 · 10^−2^ (1)
2	2	rs3755076^2^	5.18 · 10^−1^	5.30 · 10^−4^ (48)
2	17	rs11887827^3,4^	2.42 · 10^−8^	1.27 · 10^−3^ (20)
2	5	rs10210302^1,4,5^ (*ATG16L1*)	2.22 · 10^−13^	2.79 · 10^−3^ (6)
3	6	rs11718165^1,5^ (*BSN*)	1.70 · 10^−6^	1.19 · 10^−3^ (24)
4	2	rs17045935^4^ (*ANK2*)	5.28 · 10^−2^	6.45 · 10^−4^ (39)
5	3	rs16893874	3.18 · 10^−5^	3.32 · 10^−4^ (80)
5	12	rs17234657^1,4,5^	1.72 · 10^−13^	2.26 · 10^−3^ (10)
5	2	rs17149128 (*SNCAIP*)	4.10 · 10^−1^	1.97 · 10^−4^ (166)
5	4	rs931058^5^	1.53 · 10^−8^	5.83 · 10^−4^ (44)
6	2	rs600382	2.38 · 10^−5^	2.67 · 10^−4^ (95)
8	4	rs10216909^4^	7.76 · 10^−5^	3.04 · 10^−4^ (87)
10	2	rs16919914	2.22 · 10^−1^	5.20 · 10^−4^ (49)
11	2	rs1533339 (*NTM*)	2.78 · 10^−4^	2.15 · 10^−4^ (145)
16	4	rs2076756^1,4,5^ (*NOD2*)	3.95 · 10^−15^	3.88 · 10^−3^ (4)
23	2	rs6522332	3.23 · 10^−1^	2.08 · 10^−4^ (155)
7	1	rs834771^2,6^	1.25 · 10^−3^	1.91 · 10^−4^ (177)
8	1	rs10957818^2,6^	2.62 · 10^−5^	2.13 · 10^−4^ (151)
14	1	rs4903604^2,6^	2.48 · 10^−3^	2.89 · 10^−4^ (89)
18	1	rs2542151^1,5,6^	7.21 · 10^−8^	2.07 · 10^−4^ (156)
**T-Trees**				
**chr**	**size**	**rsid**	**trend p-value**	**importance**
1	2	rs12409315	2.54 · 10^−3^	4.36 · 10^−4^ (32)
1	10	rs11209026^1,4,5^ (*IL23R*)	8.24 · 10^−18^	5.23 · 10^−3^ (5)
1	2	rs11162341	8.99 · 10^−1^	2.28 · 10^−4^ (57)
1	5	rs6677092 (*RPS7P5*)	1.77 · 10^−4^	4.15 · 10^−4^ (33)
2	35	rs11887827^3,4^	2.42 · 10^−8^	1.03 · 10^−2^ (1)
2	2	SNP_A-2293058	1.79 · 10^−5^	1.81 · 10^−4^ (78)
2	5	rs10210302^1,4,5^ (*ATG16L1*)	2.22 · 10^−13^	3.07 · 10^−4^ (48)
3	2	rs17047422	3.45 · 10^−4^	1.91 · 10^−4^ (73)
3	2	rs6774 (*B4GALT4*)	1.39 · 10^−2^	3.41 · 10^−4^ (43)
3	2	rs4686733	3.65 · 10^−1^	1.39 · 10^−4^ (93)
4	2	rs1872321	6.88 · 10^−9^	1.19 · 10^−3^ (17)
4	2	rs17045935^4^ (*ANK2*)	5.28 · 10^−2^	2.57 · 10^−4^ (53)
4	3	rs1595154	1.08 · 10^−7^	5.70 · 10^−4^ (28)
5	10	rs17234657^1,4,5^	1.72 · 10^−13^	4.55 · 10^−4^ (30)
6	2	rs16884693^5^	1.21 · 10^−3^	9.36 · 10^−5^ (145)
6	3	rs2784899	6.48 · 10^−2^	1.26 · 10^−4^ (106)
7	2	rs10270692	9.31 · 10^−2^	1.99 · 10^−4^ (68)
7	9	rs6947579^3^	8.54 · 10^−1^	7.55 · 10^−3^ (3)
8	2	rs10216909^4^	7.76 · 10^−54^	1.03 · 10^−4^ (131)
10	2	rs11011417	1.85 · 10^−5^	1.31 · 10^−4^ (100)
11	2	rs9804490	2.41 · 10^−5^	1.16 · 10^−4^ (117)
12	2	rs11613902 (*TMEM117*)	9.43 · 10^−1^	3.46 · 10^−4^ (41)
14	4	rs10144260	1.18 · 10^−9^	1.07 · 10^−3^ (18)
14	2	rs2819467 (*C14orf79*)	1.51 · 10^−3^	1.23 · 10^−4^ (110)
16	3	rs2076756^1,4,5^ (*NOD2*)	3.95 · 10^−15^	6.43 · 10^−4^ (25)
23	8	rs5904497 (*SMS*)	4.41 · 10^−2^	3.26 · 10^−3^ (9)
23	2	rs6624585 (*NHSL2*)	2.69 · 10^−2^	2.24 · 10^−4^ (58)
3	1	rs11718165^1,5,6^ (*BSN*)	1.70 · 10^−6^	7.93 · 10^−5^ (159)
5	1	rs2279980^2,6^	6.19 · 10^−5^	7.03 · 10^−5^ (182)
8	1	rs10957818^2,6^	2.62 · 10^−5^	1.06 · 10^−4^ (126)
18	1	rs2542151^1,5,6^	7.21 · 10^−8^	9.35 · 10^−5^ (146)

Regions highlighted from the top 200 SNPs according to SNP importances with RF (top) and T-Trees (bottom) on *CD_qc_*. Each row corresponds to a set of SNPs obtained by merging contiguous SNPs in the rankings that are not separated by more than 20 SNPs. For readability, only groups of more than 2 SNPs appear in the tables. Markers that are isolated but reported as associated in [25] are nevertheless compiled at the bottom of both tables ^(6^ ^)^. For each region, the columns provide the chromosome number, the number of important SNPs in the region, the most important SNP in the region (and its gene name if provided by PheGenI [40]), the *p*-value of this SNP and its importance. ^(1)^ and ^(2)^: the regions reported as strongly (with a trend or a genotypic *p*-value <10^−5^) and moderately (with a trend or a genotypic *p*-value between 10^−5^ and 10^−4^) associated in [25]. ^(5)^: also reported by [37]. ^(4)^: regions identified by both RF and T-Trees. ^(3)^: the two novel regions mainly spotted by T-Trees.

In the case of RF, the region that contains the most important SNP, rs11209026, is located on chromosome 1 and contains 10 markers in total, spanning from 67.31Mb to 67.46Mb. SNP rs11209026 was reported in [25] as strongly associated to the disease and is also found in the 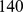 loci reported in [37]. In the SNP ranking yielded by RF, five of the WTCCC confirmed regions are selected and well represented in the 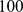 most important variables. There are ten SNPs located in the interleukin 23 receptor regions on chromosome 1, five on chromosome 2 in the ATG16L1 gene, six around rs11718165, twelve on chromosome 5 around rs17234657 and four in the NOD2 region on chromosome 16. We also notice the presence of rs2542151 alone at position 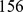 in the ranking. In addition, rs931058 on chromosome 5 has been reported in the list of 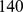 loci. We note however that this latter SNP was not reported in [25]. A few other SNPs reported only in [37] appeared isolated (and thus not reported in Table 6) in these 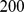 first variables: rs11260562, rs909813, rs17101358, rs10923915, rs11190083 and rs1751852.

The same five regions from the WTCCC study are also highlighted with T-Trees. In particular, rs11209026, rs10210302, rs17234657 and rs2076756 are found among the 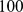 most important SNPs. The region that contains the most important SNP in the SNP ranking induced by T-Trees, rs11887827, is located on chromosome 2p12 and contains 35 SNPs among the top 200. This region was not previously identified in the literature. Part of it (i.e., 17 SNPs) is also found by RF, with the most important SNP at the 20th position in the RF ranking. The second most important region found by T-Trees is located on chromosome 7q31 and contains 9 SNPs, with the most important one, rs6947579, ranked at the third position. This region is particularly interesting as it is found neither in the literature, nor by the RF method. Additionally, rs16884693 was represented by 2 markers and a few more of the 140 loci not reported by the wtccc appeared isolated and at lower positions in the SNP ranking (rs11260562, rs17101358, rs931058, rs10772590 and rs2352937).

**Figure 2:**
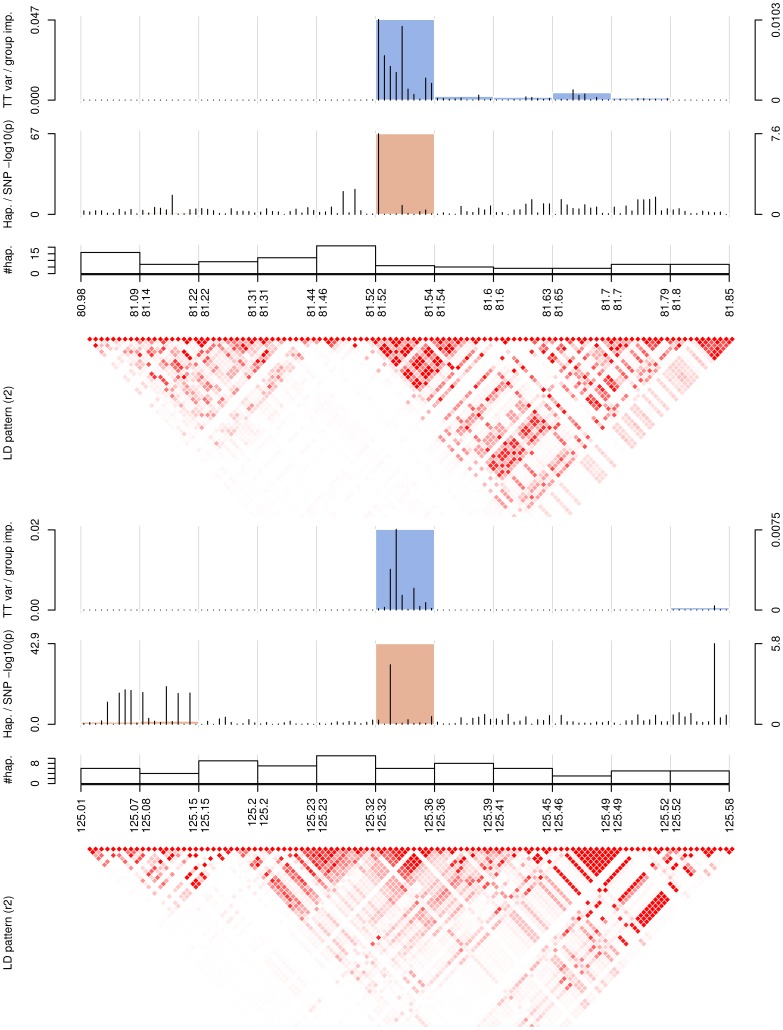
Group and variable importances for the two novel candidate regions for Crohn's disease. Regions 2p12 (top) and 7q31 (bottom), as found by T-Trees on 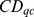. First row: SNP and block importances. Second row: univariate (Fisher) p-values and haplotype p-values as derived from the case/control omnibus test with 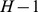 degrees of freedom where 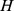 corresponds to the number of common haplotypes (a haplotype is said to be common if its frequency is greater than 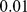 in the population under study). Third row: number of haplotypes in each block. Bottom plot: ld pattern (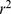) in the regions.

Figure 2 illustrates SNP and block importances (as described in Section Individual and group-wise importances), as well as single and haplotype p-values along the top two regions specifically identified with T-Trees, 2p12 (top) and 7q31 (bottom). For the 2p12 region, the plot shows that the block with the highest importance is also strongly associated with the disease. Indeed, while the univariate p-value fails to strongly identify this association (the smallest p-value in that block is 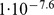), the haplotype p-value is however extremely low (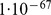). The ld pattern suggests that there are two haplotype blocks in this region, and the 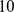 SNP block we identified with the T-Trees falls within a strongly correlated subregion in the second haplotype block. Similarly, for the 7q31 region, the same analysis shows that the corresponding block has a haplotype p-value of 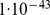 while the flanking blocks are not associated at all.

Given the low haplotype p-values found for these two blocks, they would have been also spotted by a genomewide scan of 10-SNP windows with the omnibus haplotype test. Note however that the TTrees method has found these blocks in addition to several other previously reported loci and more importantly that it directly provides a predictive model. Our method should also be more robust to the choice of the window size and irrelevant SNPs within blocks because of the tree node splitting mechanism. Trying different windows sizes with the omnibus test would very likely increase the false positive rate.

Inspection of variable importances on 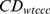 (Table S02 in Supporting Information S1) also points out that several SNPs filtered by the stronger QC filter were nevertheless considered important by both RF and TT. In particular, many of these variables are deviating from HWE. While this filter is commonly accepted as a good exclusion criteria, it is also disputed [38] as it might as well be used for the detection of marker-disease association [39]. We discovered that when such variables were exploited in a forest, they were often followed by many of their neighbors which, arguably, lowers their suspiciousness. In addition, these deviating SNPs allowed in some cases to detect signals there were not reported in the WTCCC study.

Finally, as a last comparison, we also establish a SNP ranking from the weights 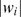 as derived from linear models (details not reported here). When considering the intersection between this ranking and those yielded by RF and T-Trees on 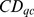, we observe the presence of several SNPs in previously reported regions (in 1p31, 2q37, 5p13 and 16q12). This intersection also includes one SNP in the 2p12 locus, i.e. one of the two regions strongly and newly identified by T-Trees. This overlap is reassuring and confirms our previous analysis. It also suggests the presence of non-linear effects in these data, since (non-linear) tree-based models indeed allow for better predictive performance than linear models, even though both detect and exploit a common subset of confirmed important SNPs.

## Discussion

Overall, due to their intrinsic multivariate and non-linear properties, tree-based ensemble methods prove to be a powerful analysis tool in the context of GWAS. In terms of risk prediction, tree-based methods show to be very effective to classify individuals given their genotypes while, in terms of loci identification, they confirm to be a well-suited alternative to standard approaches.

In this work, we proposed an extension of random forests, called T-Trees, explicitly designed to take advantage of linkage disequilibrium in GWAS data. We empirically evaluated the proposed method and compared our results with standard tree-based approaches and linear models. In all our experiments, we noted significant improvement in terms of predictive power, hence suggesting the actual existence of multivariate and/or non-linear effects due to the combination of several SNPs. Our results generalized and remained consistent across a wide range of experiments. In particular, while we found that tree-based methods may be sensitive to particular types of variables (rare variants, markers deviating from HWE), conclusions on the settings remain consistent across the seven WTCCC datasets.

In terms of identification of associated loci, tree-based methods have been able to recover most of the loci already reported in the literature, thereby confirming their relevance in this context. Most interestingly, T-Trees identified two novel susceptibility loci in the context of Crohn’s disease. By all appearances, these two regions are potentially relevant from the biological point of view, but require further experimental analysis to confirm their actual relevance.

Finally, we also noted the importance of quality control filters, which may either remove strong but associated signals or indirectly hide weaker associations if spurious signals are not filtered out.

Directions for further enhancing tree-based methods in the context of structured input variables are various. In particular, one of them would be to take explicitly into account the observed correlation structure in a given dataset when defining the blocks of variables exploited by the method. This could be achieved by combining available databases (such as HapMap) and software outputs (such as Haploview). Also, while T-Trees are currently designed for binary traits, possible extensions include quantitative non-binary traits (e.g., using regression trees). Finally, internal decision trees in the outer nodes could also be replaced by other types of weak learners.

## Supporting Information

Supporting Information S1 **Supplementary figures and tables.** T-Trees algorithm: pseudo-code and implementation details.
(PDF)
