# Supplementary material for: Exploiting SNP Correlations within Random Forest for Genome-Wide Association Studies
Source: PLoS One. 2014 Apr 2;9(4):e93379. doi: 10.1371/journal.pone.0093379 (PMC3973686; doi:10.1371/journal.pone.0093379)
Supplement: Supporting Information S1 — Supplementary figures and tables. T-Trees algorithm: pseudo-code and implementation details. (PDF) [file pone.0093379.s001.pdf]

Exploiting SNP correlations within Random Forest for Genome-Wide Association Studies
Supporting Information S1

Supplementary figures

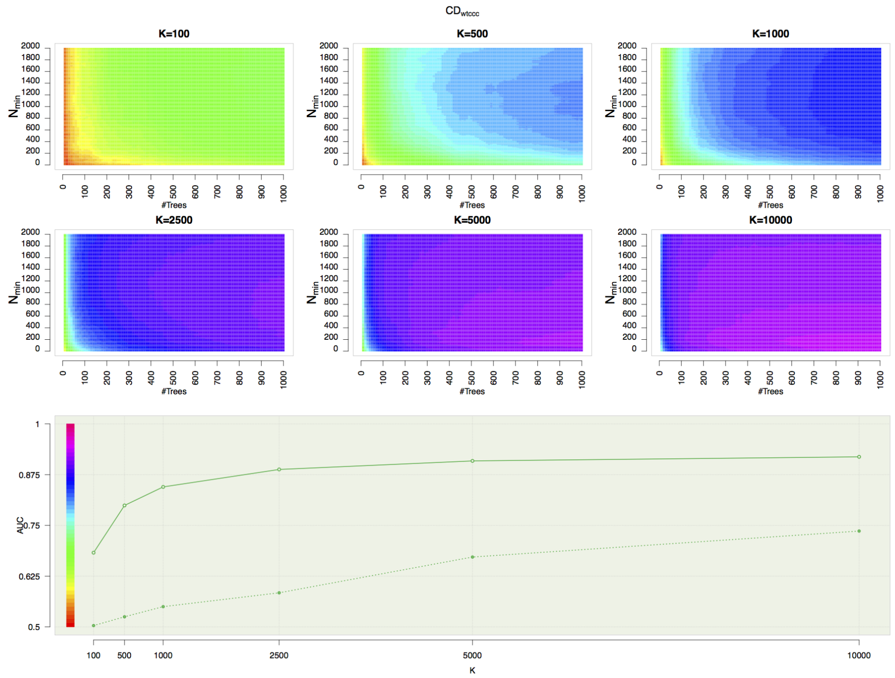

Figure S01: Random Forest: influence of T, $N_{\min}$ and K on CD_{wtccc}. The six upper panels show the influence of T and $N_{\min}$ for each of the six investigated values of K. The last panel displays the evolution of the AUC as the value of K increases. The plain (resp. dotted) line plots for each value of K the maximum (resp. minimum) AUC over all possible values of T and N_{min}.

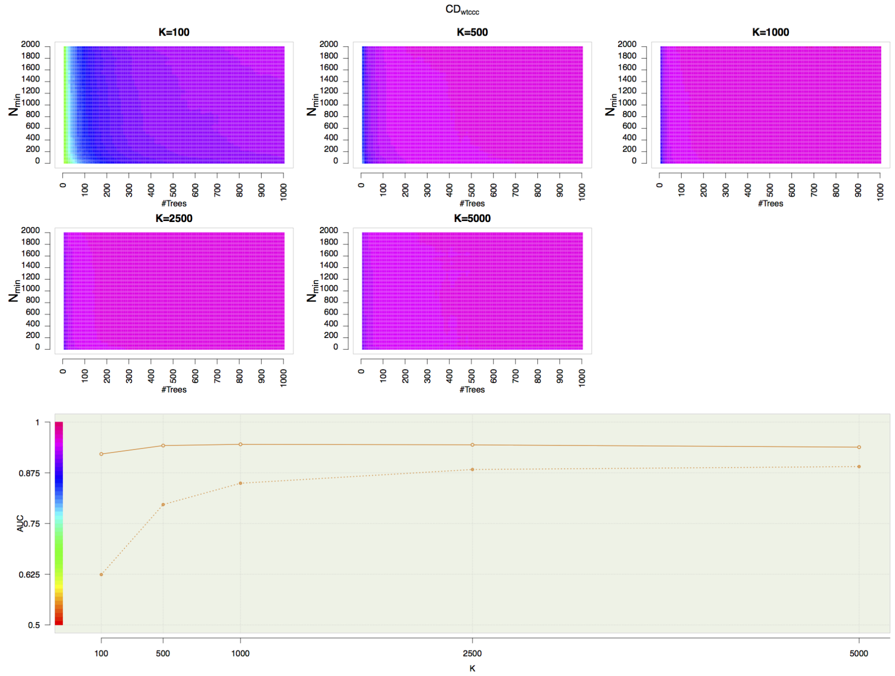

Figure S02: On CD_{wtccc}: T-Trees prediction performance with 10-SNPs blocks and $IC = 10$. The five upper panels show the influence of T and $N_{\min}$ for each of the five investigated values of K. The last panel displays the evolution of the AUC as the value of K increases. The plain (resp. dotted) line plots for each value of K the maximum (resp. minimum) AUC over all possible values of T and N_{min}.

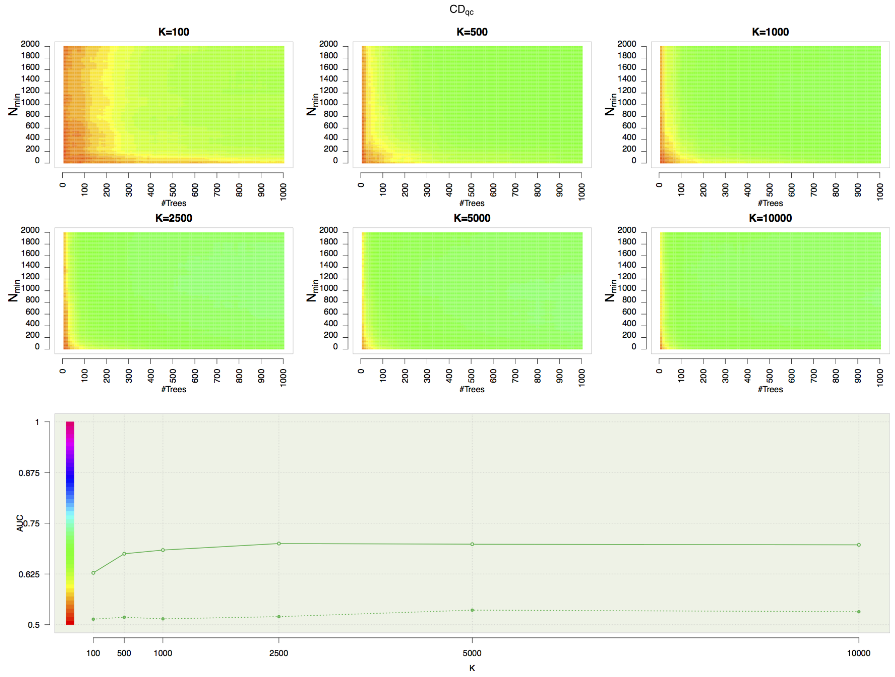

Figure S03: Random Forest: influence of T, N_{min} and K on CD_{qc}. The six upper panels show the influence of T and $N_{\min}$ for each of the six investigated values of K. The last panel displays the evolution of the AUC as the value of K increases. The plain (resp. dotted) line plots for each value of K the maximum (resp. minimum) AUC over all possible values of T and N_{min}.

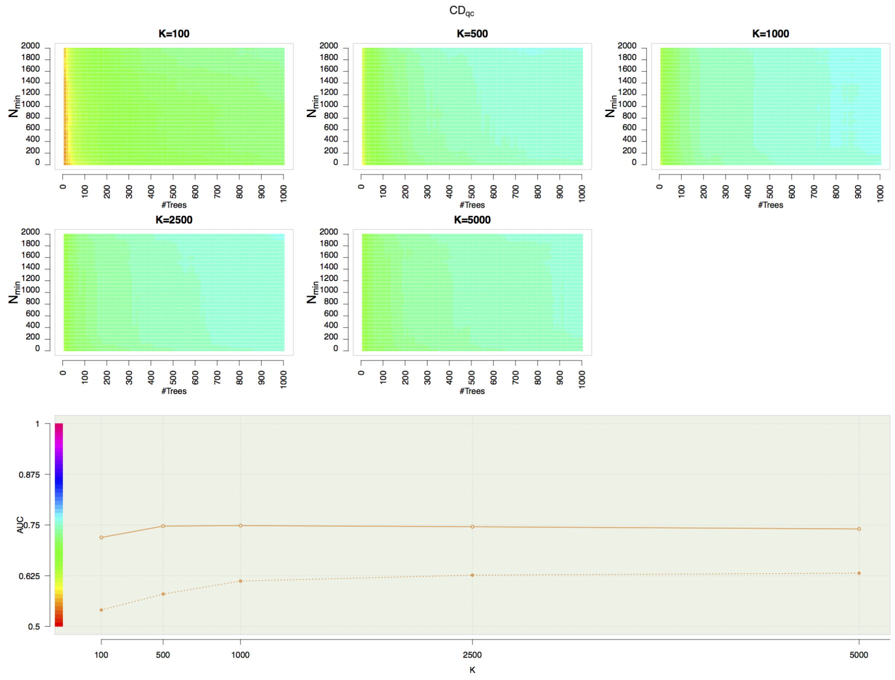

Figure S04: On CD_{qc}: T-Trees prediction performance with 10-SNPs blocks and $IC = 10$. The five upper panels show the influence of T and $N_{\min}$ for each of the five investigated values of K. The last panel displays the evolution of the AUC as the value of K increases. The plain (resp. dotted) line plots for each value of K the maximum (resp. minimum) AUC over all possible values of T and N_{min}.

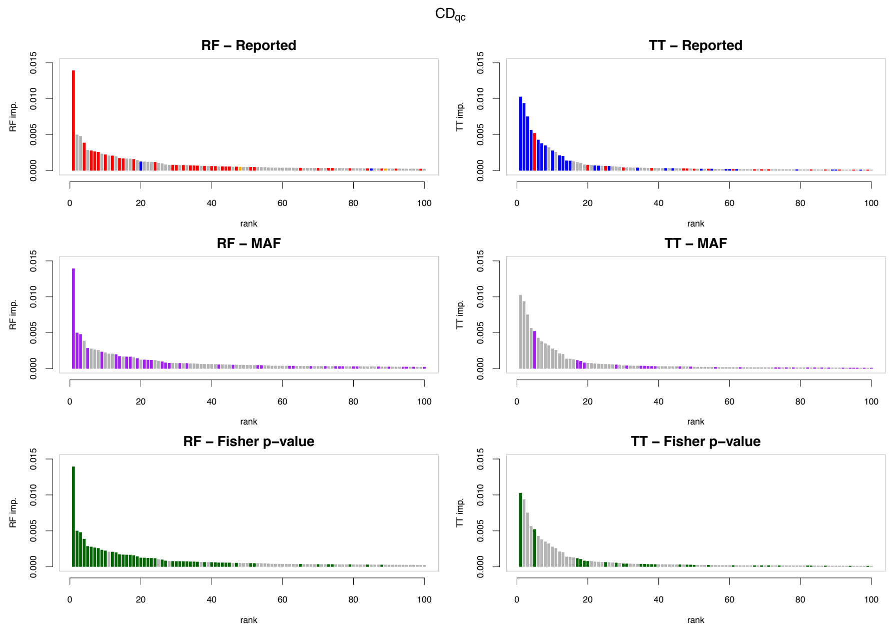

Figure S05: The first 100 variables according to the tree based importance rankings for CD_{qc}. The horizontal axis corresponds to the ranks and the vertical axis to the variable importances. In the first column variables are ordered according to random forests variable importances and in the second column they are ordered according to the T-Trees variable importances. In the first row, red highlights the nine reported regions and blue highlights two more regions mostly detected by tree based methods. In the second row, purple corresponds to rare variants (MAF < 0.05). In the third row, green represents markers with a low Fisher p-value ($< 10^{-6}$).

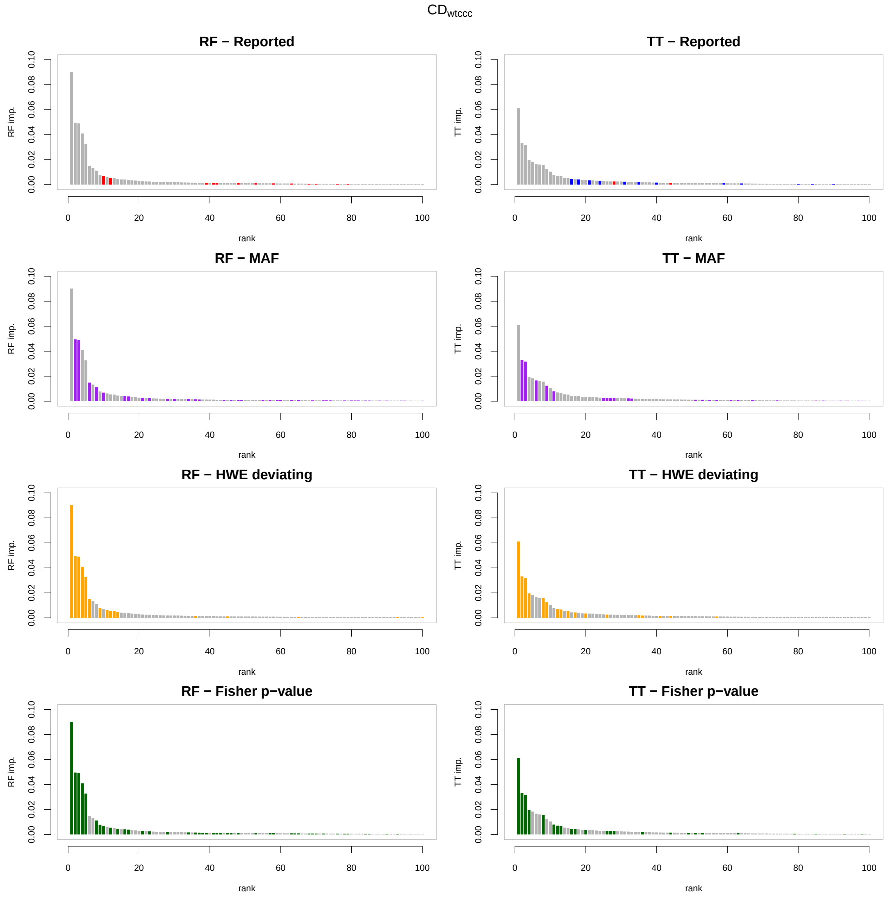

Figure S06: The first 100 variables according to the tree based importance rankings for CD_{wtccc}. The horizontal axis corresponds to the ranks and the vertical axis to the variable importances. In the first column variables are ordered according to random forests variable importances and in the second column they are ordered according to the T-Trees variable importances. In the first row, red highlights the nine reported regions and blue highlights two more regions mostly detected by tree based methods. In the second row, purple corresponds to rare variants (MAF < 0.05). In the third row, orange highlights SNPs deviating from HWE and in the last row, green represents markers with a low Fisher p-value ($< 10^{-6}$).

Supplementary tables

Random Forests

chr	start	end	size	rsid	MAF	HWE_{case}	$HWE_{control}$	p-value	importance
1	67.31	67.46	10	rs11209026 (*IL23R*)	0.045	1	$3.52 \cdot 10^{-5}$	$8.24 \cdot 10^{-18}$	$1.40 \cdot 10^{-2}$ (1)
2	45.58	45.58	2	rs3755076	0.087	$2.65 \cdot 10^{-3}$	$6.02 \cdot 10^{-3}$	$5.18 \cdot 10^{-1}$	$5.30 \cdot 10^{-4}$ (48)
2	81.58	81.76	17	rs11887827	0.311	$1.54 \cdot 10^{-7}$	1	$2.42 \cdot 10^{-8}$	$1.27 \cdot 10^{-3}$ (20)
2	233.94	233.97	5	rs10210302 (*ATG16L1*)	0.452	$4.25 \cdot 10^{-1}$	$1.98 \cdot 10^{-2}$	$2.22 \cdot 10^{-13}$	$2.79 \cdot 10^{-3}$ (6)
3	49.43	49.68	6	rs11718165 (*BSN*)	0.295	$1.33 \cdot 10^{-2}$	$1.60 \cdot 10^{-2}$	$1.70 \cdot 10^{-6}$	$1.19 \cdot 10^{-3}$ (24)
4	114.61	114.62	2	rs17045935 (*ANK2*)	0.095	$2.38 \cdot 10^{-1}$	$1.07 \cdot 10^{-4}$	$5.28 \cdot 10^{-2}$	$6.45 \cdot 10^{-4}$ (39)
5	24.77	24.77	3	rs16893874	0.008	$2.61 \cdot 10^{-1}$	1	$3.18 \cdot 10^{-5}$	$3.32 \cdot 10^{-4}$ (80)
5	40.43	40.61	12	rs17234657	0.146	$4.18 \cdot 10^{-1}$	$3.51 \cdot 10^{-1}$	$1.72 \cdot 10^{-13}$	$2.26 \cdot 10^{-3}$ (10)
5	121.75	121.76	2	rs17149128 (*SNCAIP*)	0.122	$1.10 \cdot 10^{-2}$	$1.03 \cdot 10^{-2}$	$4.10 \cdot 10^{-1}$	$1.97 \cdot 10^{-4}$ (166)
5	150.21	150.31	4	rs931058	0.071	$5.64 \cdot 10^{-1}$	1	$1.53 \cdot 10^{-8}$	$5.83 \cdot 10^{-4}$ (44)
6	36.54	36.64	2	rs600382	0.001	1	1	$2.38 \cdot 10^{-5}$	$2.67 \cdot 10^{-4}$ (95)
8	129.88	129.96	4	rs10216909	0.003	1	1	$7.76 \cdot 10^{-5}$	$3.04 \cdot 10^{-4}$ (87)
10	65.96	65.96	2	rs16919914	0.080	$8.80 \cdot 10^{-2}$	$4.00 \cdot 10^{-4}$	$2.22 \cdot 10^{-1}$	$5.20 \cdot 10^{-4}$ (49)
11	130.84	130.84	2	rs1533339 (*NTM*)	0.005	1	1	$2.78 \cdot 10^{-4}$	$2.15 \cdot 10^{-4}$ (145)
16	49.30	49.32	4	rs2076756 (*NOD2*)	0.270	$4.50 \cdot 10^{-3}$	$7.62 \cdot 10^{-1}$	$3.95 \cdot 10^{-15}$	$3.88 \cdot 10^{-3}$ (4)
23	89.59	89.64	2	rs6522332	0.160	$3.10 \cdot 10^{-1}$	$5.50 \cdot 10^{-1}$	$3.23 \cdot 10^{-1}$	$2.08 \cdot 10^{-4}$ (155)
7	135.31	135.31	1	rs834771	0.151	$1.01 \cdot 10^{-1}$	$3.37 \cdot 10^{-2}$	$1.25 \cdot 10^{-3}$	$1.91 \cdot 10^{-4}$ (177)
8	77.90	77.90	1	rs10957818	0.024	$7.13 \cdot 10^{-1}$	$6.26 \cdot 10^{-1}$	$2.62 \cdot 10^{-5}$	$2.13 \cdot 10^{-4}$ (151)
14	77.10	77.10	1	rs4903604	0.227	$5.78 \cdot 10^{-3}$	$2.41 \cdot 10^{-2}$	$2.48 \cdot 10^{-3}$	$2.89 \cdot 10^{-4}$ (89)
18	12.77	12.77	1	rs2542151	0.180	$3.08 \cdot 10^{-1}$	$9.46 \cdot 10^{-1}$	$7.21 \cdot 10^{-8}$	$2.07 \cdot 10^{-4}$ (156)

T-Trees

chr	start	end	size	rsid	MAF	HWE_{case}	$HWE_{control}$	p-value	importance
1	3.25	3.26	2	rs12409315	0.077	$1.75 \cdot 10^{-1}$	$4.37 \cdot 10^{-3}$	$2.54 \cdot 10^{-3}$	$4.36 \cdot 10^{-4}$ (32)
1	67.31	67.46	10	rs11209026 (*IL23R*)	0.045	1	$3.52 \cdot 10^{-5}$	$8.24 \cdot 10^{-18}$	$5.23 \cdot 10^{-3}$ (5)
1	77.61	77.62	2	rs11162341	0.132	$4.01 \cdot 10^{-1}$	$3.78 \cdot 10^{-1}$	$8.99 \cdot 10^{-1}$	$2.28 \cdot 10^{-4}$ (57)
1	236.50	236.50	5	rs6677092 (*RPS7P5*)	0.373	$3.01 \cdot 10^{-6}$	1	$1.77 \cdot 10^{-4}$	$4.15 \cdot 10^{-4}$ (33)
2	81.58	81.85	35	rs11887827	0.311	$1.54 \cdot 10^{-7}$	1	$2.42 \cdot 10^{-8}$	$1.03 \cdot 10^{-2}$ (1)
2	143.22	143.28	2	SNP_A-2293058	0.003	1	1	$1.79 \cdot 10^{-5}$	$1.81 \cdot 10^{-4}$ (78)
2	233.94	233.97	5	rs10210302 (*ATG16L1*)	0.452	$4.25 \cdot 10^{-1}$	$1.98 \cdot 10^{-2}$	$2.22 \cdot 10^{-13}$	$3.07 \cdot 10^{-4}$ (48)
3	7.49	7.50	2	rs17047422	0.001	1	1	$3.45 \cdot 10^{-4}$	$1.91 \cdot 10^{-4}$ (73)
3	120.41	120.42	2	rs6774 (*B4GALT4*)	0.108	$2.50 \cdot 10^{-1}$	$7.10 \cdot 10^{-4}$	$1.39 \cdot 10^{-2}$	$3.41 \cdot 10^{-4}$ (43)
3	187.31	187.35	2	rs4686733	0.053	$6.50 \cdot 10^{-5}$	$1.12 \cdot 10^{-2}$	$3.65 \cdot 10^{-1}$	$1.39 \cdot 10^{-4}$ (93)
4	86.13	86.18	2	rs1872321	0.002	1	1	$6.88 \cdot 10^{-9}$	$1.19 \cdot 10^{-3}$ (17)
4	114.61	114.62	2	rs17045935 (*ANK2*)	0.095	$2.38 \cdot 10^{-1}$	$1.07 \cdot 10^{-4}$	$5.28 \cdot 10^{-2}$	$2.57 \cdot 10^{-4}$ (53)
4	178.27	178.28	3	rs1595154	0.002	1	1	$1.08 \cdot 10^{-7}$	$5.70 \cdot 10^{-4}$ (28)
5	40.43	40.53	10	rs17234657	0.146	$4.18 \cdot 10^{-1}$	$3.51 \cdot 10^{-1}$	$1.72 \cdot 10^{-13}$	$4.55 \cdot 10^{-4}$ (30)
6	21.33	21.35	2	rs16884693	0.004	1	1	$1.21 \cdot 10^{-3}$	$9.36 \cdot 10^{-5}$ (145)
6	129.84	129.84	3	rs2784899	0.260	$8.06 \cdot 10^{-1}$	$5.25 \cdot 10^{-1}$	$6.48 \cdot 10^{-2}$	$1.26 \cdot 10^{-4}$ (106)
7	35.37	35.37	2	rs10270692	0.066	1	$6.68 \cdot 10^{-1}$	$9.31 \cdot 10^{-2}$	$1.99 \cdot 10^{-4}$ (68)
7	125.13	125.16	9	rs6947579	0.317	$2.45 \cdot 10^{-1}$	$7.02 \cdot 10^{-1}$	$8.54 \cdot 10^{-1}$	$7.55 \cdot 10^{-3}$ (3)
8	129.90	129.92	2	rs10216909	0.003	1	1	$7.76 \cdot 10^{-5}$	$1.03 \cdot 10^{-4}$ (131)
10	38.31	38.38	2	rs11011417	0.001	1	1	$1.85 \cdot 10^{-5}$	$1.31 \cdot 10^{-4}$ (100)
11	14.16	14.16	2	rs9804490	0.459	$1.50 \cdot 10^{-10}$	$1.44 \cdot 10^{-6}$	$2.41 \cdot 10^{-5}$	$1.16 \cdot 10^{-4}$ (117)
12	42.78	42.80	2	rs11613902 (*TMEM117*)	0.099	$3.98 \cdot 10^{-7}$	$9.76 \cdot 10^{-2}$	$9.43 \cdot 10^{-1}$	$3.46 \cdot 10^{-4}$ (41)
14	84.39	84.43	4	rs10144260	0.008	1	1	$1.18 \cdot 10^{-9}$	$1.07 \cdot 10^{-3}$ (18)
14	104.47	104.53	2	rs2819467 (*C14orf79*)	0.011	$3.21 \cdot 10^{-1}$	1	$1.51 \cdot 10^{-3}$	$1.23 \cdot 10^{-4}$ (110)
16	49.30	49.31	3	rs2076756 (*NOD2*)	0.270	$4.50 \cdot 10^{-3}$	$7.62 \cdot 10^{-1}$	$3.95 \cdot 10^{-15}$	$6.43 \cdot 10^{-4}$ (25)
23	21.69	21.74	8	rs5904497 (*SMS*)	0.273	$5.97 \cdot 10^{-6}$	$1.50 \cdot 10^{-1}$	$4.41 \cdot 10^{-2}$	$3.26 \cdot 10^{-3}$ (9)
23	70.94	70.94	2	rs6624585 (*NHSL2*)	0.068	$7.76 \cdot 10^{-1}$	1	$2.69 \cdot 10^{-2}$	$2.24 \cdot 10^{-4}$ (58)
3	49.67	49.67	1	rs11718165 (*BSN*)	0.295	$1.33 \cdot 10^{-2}$	$1.60 \cdot 10^{-2}$	$1.70 \cdot 10^{-6}$	$7.93 \cdot 10^{-5}$ (159)
5	57.95	57.95	1	rs2279980	0.188	$8.16 \cdot 10^{-2}$	$7.99 \cdot 10^{-1}$	$6.19 \cdot 10^{-5}$	$7.03 \cdot 10^{-5}$ (182)
8	77.90	77.90	1	rs10957818	0.024	$7.13 \cdot 10^{-1}$	$6.26 \cdot 10^{-1}$	$2.62 \cdot 10^{-5}$	$1.06 \cdot 10^{-4}$ (126)
18	12.77	12.77	1	rs2542151	0.180	$3.08 \cdot 10^{-1}$	$9.46 \cdot 10^{-1}$	$7.21 \cdot 10^{-8}$	$9.35 \cdot 10^{-5}$ (146)

Table S01: Variable importances analysis on CD_{qc}. List of regions identified by the Random Forest (upper table) and the T-Trees (bottom table). In red and orange, the regions reported as strongly, resp. moderately, associated by the [WTC07] (Supplementary Information). Underlined when reported by [JRW+13]. The gray shaded rows corresponds to regions identified by both tree-based methods. And in blue, the two novel regions mainly spotted with the T-Trees approach.

Random Forests

chr	start	end	size	rsid	MAF	HWE_{case}	$HWE_{control}$	p-value	importance
1	67.31	67.46	11	rs11209026 (*IL23R*)	0.045	1	$3.53 \cdot 10^{-5}$	$5.43 \cdot 10^{-18}$	$6.89 \cdot 10^{-3}$ (10)
1	117.16	117.18	9	rs12078461 (*PTGFRN*)	0.047	$2.51 \cdot 10^{-182}$	$4.91 \cdot 10^{-2}$	$7.19 \cdot 10^{-13}$	$4.95 \cdot 10^{-2}$ (2)
2	25.31	25.36	3	rs2164411	0.155	$2.85 \cdot 10^{-13}$	$1.95 \cdot 10^{-1}$	$4.82 \cdot 10^{-3}$	$5.32 \cdot 10^{-3}$ (13)
2	233.94	233.97	5	rs10210302 (*ATG16L1*)	0.451	$4.26 \cdot 10^{-1}$	$1.98 \cdot 10^{-2}$	$1.08 \cdot 10^{-13}$	$1.32 \cdot 10^{-3}$ (41)
3	16.45	16.46	2	rs9839841 (*RFTN1*)	0.195	$2.30 \cdot 10^{-6}$	$5.14 \cdot 10^{-1}$	$7.20 \cdot 10^{-13}$	$1.38 \cdot 10^{-3}$ (38)
3	49.43	49.68	3	rs11718165 (*BSN*)	0.295	$1.57 \cdot 10^{-2}$	$1.61 \cdot 10^{-2}$	$2.21 \cdot 10^{-6}$	$4.21 \cdot 10^{-4}$ (102)
4	16.37	16.48	14	rs157613 (*LDB2*)	0.082	$3.16 \cdot 10^{-253}$	$3.96 \cdot 10^{-6}$	$1.13 \cdot 10^{-14}$	$9.02 \cdot 10^{-2}$ (1)
4	17.73	17.93	16	rs1553460	0.315	$5.84 \cdot 10^{-93}$	$2.88 \cdot 10^{-5}$	$1.59 \cdot 10^{-31}$	$4.09 \cdot 10^{-2}$ (4)
4	158.43	158.43	3	rs17035797 (*GLRB*)	0.074	$1.38 \cdot 10^{-10}$	$9.06 \cdot 10^{-1}$	$1.29 \cdot 10^{-9}$	$5.16 \cdot 10^{-4}$ (93)
4	186.09	186.11	3	rs13126272 (*ACSL1*)	0.338	$5.00 \cdot 10^{-58}$	$3.02 \cdot 10^{-2}$	$3.65 \cdot 10^{-5}$	$6.24 \cdot 10^{-3}$ (11)
5	40.37	40.52	9	rs17234657	0.146	$4.18 \cdot 10^{-1}$	$3.52 \cdot 10^{-1}$	$2.37 \cdot 10^{-13}$	$7.16 \cdot 10^{-4}$ (68)
5	117.00	117.07	16	rs2416472	0.319	$2.40 \cdot 10^{-17}$	$9.35 \cdot 10^{-1}$	$7.19 \cdot 10^{-12}$	$4.55 \cdot 10^{-3}$ (14)
6	121.66	121.68	3	rs17083420 (*C6orf170*)	0.009	$3.45 \cdot 10^{-11}$	1	$7.68 \cdot 10^{-7}$	$1.52 \cdot 10^{-3}$ (36)
9	132.51	132.60	13	rs302925	0.475	$4.67 \cdot 10^{-20}$	$5.50 \cdot 10^{-1}$	$6.32 \cdot 10^{-6}$	$1.15 \cdot 10^{-3}$ (45)
10	125.67	125.67	3	rs7067790	0.391	$4.18 \cdot 10^{-22}$	$3.56 \cdot 10^{-1}$	$1.10 \cdot 10^{-7}$	$7.82 \cdot 10^{-3}$ (9)
11	113.02	113.31	17	rs17116117 (*HTR3B*)	0.049	$2.92 \cdot 10^{-4}$	$1.16 \cdot 10^{-1}$	$1.03 \cdot 10^{-23}$	$1.12 \cdot 10^{-2}$ (8)
14	97.06	97.07	6	rs234202	0.008	1	1	$1.21 \cdot 10^{-9}$	$1.41 \cdot 10^{-3}$ (37)
16	30.23	30.29	3	rs4471699 (*LOC595101*)	0.449	$2.31 \cdot 10^{-49}$	$6.03 \cdot 10^{-1}$	$4.64 \cdot 10^{-20}$	$3.27 \cdot 10^{-2}$ (5)
16	49.30	49.31	3	rs2076756 (*NOD2*)	0.270	$4.61 \cdot 10^{-3}$	$7.62 \cdot 10^{-1}$	$3.00 \cdot 10^{-15}$	$1.38 \cdot 10^{-3}$ (39)
2	45.58	45.58	1	rs3755076	0.087	$2.76 \cdot 10^{-3}$	$6.02 \cdot 10^{-3}$	$4.95 \cdot 10^{-1}$	$1.52 \cdot 10^{-4}$ (200)
6	32.79	32.79	1	rs3104404	0.148	$5.65 \cdot 10^{-2}$	$1.03 \cdot 10^{-1}$	$2.31 \cdot 10^{-6}$	$2.21 \cdot 10^{-4}$ (155)

T-Trees

chr	start	end	size	rsid	MAF	HWE_{case}	$HWE_{control}$	p-value	importance
1	67.31	67.42	5	rs11209026 (*IL23R*)	0.045	1	$3.53 \cdot 10^{-5}$	$5.43 \cdot 10^{-18}$	$2.55 \cdot 10^{-3}$ (28)
1	117.16	117.18	10	rs12078461 (*PTGFRN*)	0.047	$2.51 \cdot 10^{-182}$	$4.91 \cdot 10^{-2}$	$7.19 \cdot 10^{-13}$	$3.32 \cdot 10^{-2}$ (2)
1	214.81	214.86	6	rs1933641 (*RRP15*)	0.047	$3.76 \cdot 10^{-180}$	$7.90 \cdot 10^{-2}$	$7.59 \cdot 10^{-14}$	$3.17 \cdot 10^{-2}$ (3)
2	25.31	25.36	3	rs2164411	0.155	$2.85 \cdot 10^{-13}$	$1.95 \cdot 10^{-1}$	$4.82 \cdot 10^{-3}$	$1.53 \cdot 10^{-3}$ (41)
2	81.54	81.72	14	rs11887827	0.311	$1.66 \cdot 10^{-7}$	1	$2.19 \cdot 10^{-8}$	$4.37 \cdot 10^{-3}$ (16)
2	132.48	132.55	2	rs4080478	0.104	$2.69 \cdot 10^{-8}$	$1.44 \cdot 10^{-4}$	$3.64 \cdot 10^{-3}$	$9.92 \cdot 10^{-4}$ (62)
3	16.45	16.46	2	rs9839841 (*RFTN1*)	0.195	$2.30 \cdot 10^{-6}$	$5.14 \cdot 10^{-1}$	$7.20 \cdot 10^{-13}$	$4.83 \cdot 10^{-4}$ (93)
3	18.60	18.60	2	rs12714959	0.263	$1.74 \cdot 10^{-23}$	$7.82 \cdot 10^{-5}$	$5.06 \cdot 10^{-7}$	$1.46 \cdot 10^{-3}$ (44)
3	184.57	184.57	3	rs959880 (*MCF2L2*)	0.144	$4.85 \cdot 10^{-1}$	$2.65 \cdot 10^{-1}$	$4.64 \cdot 10^{-1}$	$1.14 \cdot 10^{-3}$ (56)
4	16.43	16.48	15	rs157613 (*LDB2*)	0.082	$3.16 \cdot 10^{-253}$	$3.96 \cdot 10^{-6}$	$1.13 \cdot 10^{-14}$	$6.11 \cdot 10^{-2}$ (1)
4	17.85	17.90	9	rs1553460	0.315	$5.84 \cdot 10^{-93}$	$2.88 \cdot 10^{-5}$	$1.59 \cdot 10^{-31}$	$1.96 \cdot 10^{-2}$ (4)
4	38.77	38.78	2	rs6816863	0.027	$4.18 \cdot 10^{-89}$	1	$2.86 \cdot 10^{-1}$	$1.25 \cdot 10^{-2}$ (9)
4	56.95	56.96	2	rs4865080 (*KIAA1211*)	0.002	1	1	$3.98 \cdot 10^{-10}$	$5.48 \cdot 10^{-4}$ (85)
4	158.43	158.43	3	rs17035814 (*GLRB*)	0.089	$1.05 \cdot 10^{-1}$	$6.44 \cdot 10^{-1}$	$7.35 \cdot 10^{-1}$	$2.52 \cdot 10^{-3}$ (29)
4	186.09	186.13	6	rs13126272 (*ACSL1*)	0.338	$5.00 \cdot 10^{-58}$	$3.02 \cdot 10^{-2}$	$3.65 \cdot 10^{-5}$	$5.33 \cdot 10^{-3}$ (15)
5	40.37	40.44	2	rs1186661	0.133	$3.80 \cdot 10^{-1}$	$2.76 \cdot 10^{-3}$	$1.55 \cdot 10^{-12}$	$2.27 \cdot 10^{-4}$ (140)
5	117.02	117.06	9	rs17411921	0.339	$2.19 \cdot 10^{-1}$	$3.66 \cdot 10^{-1}$	$7.87 \cdot 10^{-1}$	$1.83 \cdot 10^{-2}$ (5)
6	93.79	93.82	10	rs6454931	0.279	$2.16 \cdot 10^{-1}$	$8.90 \cdot 10^{-1}$	$5.84 \cdot 10^{-1}$	$3.58 \cdot 10^{-3}$ (19)
6	121.63	121.68	4	rs17083420 (*C6orf170*)	0.009	$3.45 \cdot 10^{-11}$	1	$7.68 \cdot 10^{-7}$	$2.62 \cdot 10^{-3}$ (26)
7	38.93	38.97	3	rs1525791 (*POU6F2*)	0.152	$1.22 \cdot 10^{-3}$	$6.26 \cdot 10^{-1}$	$2.14 \cdot 10^{-8}$	$6.11 \cdot 10^{-4}$ (79)
7	125.13	125.14	6	rs6947579	0.317	$2.24 \cdot 10^{-1}$	$7.34 \cdot 10^{-1}$	$8.19 \cdot 10^{-1}$	$3.41 \cdot 10^{-3}$ (21)
9	132.59	132.60	7	rs10901198 (*GTF3C4*)	0.119	$9.10 \cdot 10^{-1}$	$4.79 \cdot 10^{-1}$	$7.92 \cdot 10^{-1}$	$1.04 \cdot 10^{-2}$ (10)
10	10.32	10.32	2	rs2151595	0.068	1	$7.76 \cdot 10^{-1}$	$2.60 \cdot 10^{-1}$	$3.16 \cdot 10^{-4}$ (118)
10	125.66	125.67	4	rs769282	0.415	$6.57 \cdot 10^{-1}$	$4.94 \cdot 10^{-1}$	$7.95 \cdot 10^{-1}$	$5.50 \cdot 10^{-3}$ (14)
11	55.32	55.35	2	rs7951100	0.070	$2.58 \cdot 10^{-1}$	$3.97 \cdot 10^{-1}$	$6.75 \cdot 10^{-1}$	$3.03 \cdot 10^{-4}$ (121)
11	113.28	113.31	5	rs1176741 (*HTR3B*)	0.030	$6.45 \cdot 10^{-1}$	$1.19 \cdot 10^{-1}$	$3.50 \cdot 10^{-1}$	$1.66 \cdot 10^{-2}$ (6)
14	35.06	35.16	4	rs10483456 (*RALGAPA1*)	0.059	$5.04 \cdot 10^{-1}$	$5.22 \cdot 10^{-1}$	$2.81 \cdot 10^{-11}$	$1.29 \cdot 10^{-3}$ (49)
14	59.81	59.83	3	rs7154773 (*PPM1A*)	0.352	$2.10 \cdot 10^{-1}$	$1.96 \cdot 10^{-1}$	$6.84 \cdot 10^{-1}$	$6.87 \cdot 10^{-4}$ (72)
14	83.04	83.06	2	rs10144243	0.005	1	1	$2.25 \cdot 10^{-12}$	$1.24 \cdot 10^{-3}$ (51)
14	97.06	97.10	6	rs11846702	0.012	1	$3.33 \cdot 10^{-1}$	$4.42 \cdot 10^{-1}$	$2.76 \cdot 10^{-3}$ (25)
16	29.84	30.29	7	rs11644392 (*LOC595101*)	0.484	$6.99 \cdot 10^{-1}$	$6.83 \cdot 10^{-1}$	$2.20 \cdot 10^{-1}$	$1.60 \cdot 10^{-2}$ (7)
17	50.27	50.29	2	rs2934884	0.198	$4.06 \cdot 10^{-1}$	$4.16 \cdot 10^{-1}$	$9.15 \cdot 10^{-1}$	$7.33 \cdot 10^{-4}$ (70)
23	0.63	0.64	6	rs5988334	0.216	$2.36 \cdot 10^{-2}$	$2.06 \cdot 10^{-3}$	$1.13 \cdot 10^{-5}$	$1.36 \cdot 10^{-3}$ (47)
23	2.58	2.58	2	rs1419930	0.041	$5.51 \cdot 10^{-2}$	$5.71 \cdot 10^{-19}$	$2.33 \cdot 10^{-1}$	$1.11 \cdot 10^{-3}$ (57)
23	21.73	21.73	2	rs4824171 (*SMS*)	0.284	$6.52 \cdot 10^{-1}$	$2.14 \cdot 10^{-1}$	$3.22 \cdot 10^{-1}$	$8.84 \cdot 10^{-4}$ (66)
2	233.94	233.94	1	rs10210302 (*ATG16L1*)	0.451	$4.26 \cdot 10^{-1}$	$1.98 \cdot 10^{-2}$	$1.08 \cdot 10^{-13}$	$2.68 \cdot 10^{-4}$ (132)
16	49.31	49.31	1	rs2076756 (*NOD2*)	0.270	$4.61 \cdot 10^{-3}$	$7.62 \cdot 10^{-1}$	$3.00 \cdot 10^{-15}$	$4.29 \cdot 10^{-4}$ (105)

Table S02: Variable importances analysis on CD_{wtccc}. List of regions identified by the Random Forest (upper table) and the T-Trees (bottom table). In red and orange, the regions reported as strongly, resp. moderately, associated by the [WTC07] (Supplementary Information). Underlined when reported by [JRW+13]. The gray shaded rows corresponds to regions identified by both tree-based methods. And in blue, the two novel regions mainly spotted with the T-Trees approach.

Supplementary methods

T-Trees Algorithms

```
T-Trees
input : LS,B,T,K,K_int,IC,N_min
output: 𝒯

for i ← 1 to T do
    LS_i ← Bootstrap(LS)
    T_i ← BuildTTree(LS_i,B,K,K_int,IC,N_min)
    Append(𝒯,T_i)
return 𝒯
```

Algorithm 1: The T-Trees algorithm is quite similar to the random forest algorithm. It adds two metaparameters: K_{int} and IC; and needs a block map $\mathcal{B}$.

```
BuildTTree
input : LS,B,K,K_int,IC,N_min
output: A TTree T                      // Its root node

if const(attributes) or const(output) or #LS ≥ N_min then
    return a leaf labeled by class frequencies in LS
else
    Select K random blocks ∈ B : {g_1, ..., g_K}
    {s_1, ..., s_K} : s_i = pickGroupSplit(LS, g_i, K_int, IC)
    s_* = max_{i=1,...,K} Score(s_i, LS)
    Split LS into LS_left and LS_right according to s_*
    T_right ← BuildTTree(LS_right,B,K,K_int,IC)
    T_left ← BuildTTree(LS_left,B,K,K_int,IC)
    return createNode(s_*,T_left,T_right)
```

Algorithm 2: The T-Tree building algorithm

```
pickGroupSplit
input : LS, g, K_int, IC
output: [p < th]

T = BuildExtraTTree(LS,g,K_int,IC)
Propagate LS in T, p = vector of resulting probabilities
Search optimal threshold th over p
return [p < th]
```

Algorithm 3: The `pickGroupSplit` function is based on the Extra-Trees algorithm. A single Extra-Tree is built and its predictions allow to transform a group of attributes into a new numerical value.

```
BuildExtraTTree
input  : LS,g,K_int,IC
output: A tree : t                        // Its root node
```

if *const(attributes)* **or** *const(output)* **or** $\#nodes \leq IC$ **then**
  return a leaf labeled by class frequencies in $\mathcal{LS}$
else
  Select K_{int} random attributes $\in g$: $\{a_1, ..., a_{K_{int}}\}$
  $\{s_1, ..., s_{K_{int}}\} : s_i = \texttt{pickRandomSplit}(\mathcal{LS}, a_i)$
  $s_* = max_{i=1,...,K_{int}} \texttt{Score}(s_i, \mathcal{LS})$
  Split $\mathcal{LS}$ into $\mathcal{LS}_{left}$ and $\mathcal{LS}_{right}$ according to s_*
  $T_{right} \leftarrow \texttt{BuildExtraTTree}(\mathcal{LS}_{right}, g, K_{int}, IC)$
  $T_{left} \leftarrow \texttt{BuildExtraTTree}(\mathcal{LS}_{left}, g, K_{int}, IC)$
  return $\texttt{createNode}(s_*, T_{left}, T_{right})$

Algorithm 4: Inside the outer nodes, the weak learner that is used is a single Extra-Tree with an IC-limited number of (inner) test nodes.

Implementation details

Evaluation of the splits

The score measures we used in our experiments are based on the well-known logarithmic or *Shannon* entropy. Let t denote a test outcome at a *node* of a decision tree and c the class which we are trying to predict, t and c are two discrete random variables of respective distribution $(p(t_1), ..., p(t_k))$ and $(p(c_1), ..., p(c_m))$ (in our case, as from a machine learning point of view a GWAS is a binary classification problem and the decision trees are binary trees, $m = k = 2$). Basically, the class entropy allows to measure the impurity at a given *node*:

$$\mathbf{H}_C(node) \triangleq -\sum_{i=1}^{m} p(c_i)\log_2 p(c_i) \quad (1)$$

$$\triangleq -p_{case}\log_2 p_{case} \quad (2)$$

$$-p_{control}\log_2 p_{control} \quad (3)$$

where p_{case} (where $p_{control}$) correspond to the proportion of cases (controls) reaching the current *node*. Similarly the test entropy is defined as follows:

$$\mathbf{H}_T(node) \triangleq -\sum_{j=1}^{k} p(t_j)\log_2 p(t_j) \quad (4)$$

$$\triangleq -p_{left}\log_2 p_{left} \quad (5)$$

$$-p_{right}\log_2 p_{right} \quad (6)$$

where p_{left} (p_{rigth}) denotes the proportion of objects propagated to the left (right) at the current test node. Also we can define the average conditional entropy of the class given the test:

$$\mathbf{H}_{C|T}(node) \triangleq -\sum_{i=1}^{m}\sum_{j=1}^{k} p(c_i, t_j)\log_2 p(c_i|t_j) \quad (7)$$

Thus, a score measure can be defined as follow:

$$\mathbf{score}(node) \triangleq \mathbf{H}_C(node) \quad (8)$$

$$-p_{left}\mathbf{H}_C(node_{left}) \quad (9)$$

$$-p_{right}\mathbf{H}_C(node_{right}) \quad (10)$$

$$\triangleq \mathbf{H}_C(node) - \mathbf{H}_{C|T}(node) \quad (11)$$

$$\triangleq \mathbf{I}_C^T(node) \quad (12)$$

and corresponds to the difference of the current node impurity and the weighted impurity of the two resulting child nodes. It reflects the goal of the tree induction which aims to reduce the impurity at each test node. The split that maximizes such score is the one that reduces the more the class entropy from one node to its descendants. It is also called the mutual information I_C^T and it quantifies the reduction of the uncertainty of c given t. As this information quantity is upper bounded by the prior entropy $\mathbf{H}_C(node)$, that measure is sensitive to the number and prior distribution of classes rendering it difficult to interpret. Also in the context of decision tree induction, it has been observed to favor tests at a node with a larger number of outcomes. For these reasons, various normalizations have been introduced and are discussed in details in [Weh96]. Among these, we do not recommend using the "gain ratio" as it suffers from the "end-cut" preference ([Tor01]) which we observed to be biased towards low minor allele frequency SNPs. Due to its symmetrical (in C and T) properties, we choose to use the following normalisation for our score measure:

$$\mathbf{S}_C^T \triangleq \frac{2\mathbf{I}_C^T}{(\mathbf{H}_C + \mathbf{H}_T)} \tag{13}$$

Complexity control

In some situations, it may be useful to prevent a node from being further splitted. In our tree-based methods, we use two types of complexity control parameters:

- N_{min}: this (user defined) number corresponds to the required minimum number of objects (i.e. local sub-sample size) reaching a node for it to continue splitting. For example, setting N_{min} to $n-1$ will produce a one-level decision tree (a.k.a. a *decision stump*). Practically, a simple condition is added to check whether or not the learning set is big enough to create a new split. The typical default value for N_{min} is 2; meaning that the tree is fully developed.
- N_{node}: this limit corresponds to the maximum number of test nodes allowed in a tree. Similarly, setting N_{node} to 1 will produce a decision stump. In our T-Trees method, this parameter, also referred to as the *internal complexity*: IC, is used to limit the number of inner nodes in the *weak learners*.

Labeling the leaves

We keep in the leaves the proportion of objects of each class that reached that terminal node. That proportion somehow reflect the confidence of the corresponding prediction. For example, an object reaching a terminal node with 98 cases and only 2 controls is more susceptible to be classified as a case than another object that arrives in a leaf with 32 cases and 25 controls. We use the T predictions of the T trees in a forest, they are aggregated following a "soft" voting approach in which the prediction become the average class-probability. An object is propagated into each tree, leading that object to T different leaves. The probability vectors $\mathbf{v}_i$ associated to these T leaves are averaged as follows:

$$\mathbf{v} = \frac{1}{T}\sum_{i=1}^{T}\mathbf{v}_i \tag{14}$$

and the prediction for the propagated object becomes the average class-probability $\mathbf{v}$. This aggregation method is also referred to as the 'soft' class probability aggregation. In the binary classification case, the resulting predictions $\mathbf{v} = (c_0, c_1)$ where c_0 (reps. c_1) corresponds to the probability of being classified as an object of class 0 (resp. class 1) and $c_1 = 1 - c_0$.

Variable importances

We use the mutual information for that purpose. For each variable x_i used (maybe more than once) in a decision tree, we compute its importance as follow:

$$\mathbf{V}_{imp}(x_i) = \sum_{n \in Nodes(x_i)} p_n \mathbf{I}_C^T(n) \tag{15}$$

where $Nodes(x_i)$ is the set of tree nodes where the variable x_i is used to split, p_n denotes the relative sample size of node n, and $\mathbf{I}_C^T(n)$ is the local reduction of entropy resulting from the selected split at this node.

Doing so, variables appearing in many and "bigger" nodes (i.e. closer to the root) should be more important than the other ones.

Similarly, variables importances are computed for tree ensembles by using the mutual information. In Equation 15, instead of looking at nodes from a single tree, the set $Nodes(x_i)$ becomes now the set of all nodes in the forest of T trees where variable x_i is used.

As such, this measure is however dependent on the number of trees T of the ensemble, and on the initial impurity of the dataset and the impurity reduction yielded by the trees, and is thus difficult to interpret. Hence we choose the following normalization, so as to sum up all the variable importances to 1:

$$\mathbf{V}^1_{imp}(x_i) = \frac{\mathbf{V}_{imp}(x_i)}{\sum_{i=1}^{p} \mathbf{V}_{imp}(x_i)}, \tag{16}$$

References

[JRW+13] Luke Jostins, Stephan Ripke, Rinse K Weersma, Richard H Duerr, Dermot P McGovern, et al., *Host-microbe interactions have shaped the genetic architecture of inflammatory bowel disease*, Nature **490** (2013), no. 7422, 119–124.

[Tor01] Luís Torgo, *A Study on End-Cut Preference in Least Squares Regression Trees*, EPIA '01: Proceedings of the10th Portuguese Conference on Artificial Intelligence on Progress in Artificial Intelligence, Knowledge Extraction, Multi-agent Systems, Logic Programming and Constraint Solving, Springer-Verlag, December 2001.

[Weh96] Louis Wehenkel, *On uncertainty measures used for decision tree induction*, Proceedings of the International Congress on Information Processing and Management of Uncertainty in Knowledge based Systems, IPMU96 (Granada), 1996, pp. 413–418.

[WTC07] WTCCC, *Genome-wide association study of 14,000 cases of seven common diseases and 3,000 shared controls*, Nature **447** (2007), no. 7145, 661–78.
